# ASSOCIATIONS BETWEEN SENSE OF PURPOSE, NEGATIVE AFFECT, AND CORTISOL: EVIDENCE FROM REPEATED DAILY ASSESSMENTS

**DOI:** 10.1093/geroni/igad104.3197

**Published:** 2023-12-21

**Authors:** Nathan Lewis, Theresa Pauly, Maureen Ashe, Kenneth Madden, Denis Gerstorf, Christiane Hoppmann

**Affiliations:** The University of British Columbia, Vancouver, British Columbia, Canada; Simon Fraser University, Vancouver, British Columbia, Canada; The University of British Columbia, Vancouver, British Columbia, Canada; The University of British Columbia, Vancouver, British Columbia, Canada; Humboldt University of Berlin, Berlin, Berlin, Germany; The University of British Columbia, Vancouver, British Columbia, Canada

## Abstract

A growing literature suggests that having a sense of purpose in life is key to supporting optimal aging outcomes. One proposed mechanism for such benefits posits that having strong sense of purpose facilitates better management of and less reactivity to daily negative experiences, which over time may impact health. However, there remains a paucity of research investigating sense of purpose, affective experiences, and biological stress markers as individuals engage in their daily life routines. The present study sought to examine everyday associations between sense of purpose in life, negative affect, and salivary cortisol using repeated daily life assessments. A sample of 170 older adults aged 60 to 87 (M =71.18 years, SD=6.06) completed affect ratings and provided concurrent salivary cortisol samples 4 times per day over 7 consecutive days. Multilevel growth modeling was used to examine whether trait sense of purpose in life moderated within-day associations between negative affect and log-transformed cortisol levels (nmol/L), adjusting for relevant demographic information and factors that may impact cortisol (e.g., time since waking, caffeine intake). Analyses revealed that sense of purpose was indeed associated with lower daily negative affect, but it did not predict everyday cortisol and was also not moderating the within-person couplings between everyday negative affect and cortisol. Our results suggest that purpose in life shapes daily affect dynamics of older adults, yet not through modulating the physiological implications of negative affect. Follow-up analyses will explore whether these findings generalize to vulnerable groups of very old adults and those in poor health.

